# Lavender protects H9c2 cardiomyocytes against oxygen-glucose deprivation (OGD)-induced injury via targeting the JAK2/STAT3 pathway

**DOI:** 10.22038/IJBMS.2022.54751.12280

**Published:** 2022-02

**Authors:** Shaghayegh Askarian-Amiri, Hoda Fotovat Eskandari, Fatemeh Ramezani, Gelare Vahabzadeh, Nahid Aboutaleb

**Affiliations:** 1 Institut de Biologie Structurale (IBS), Univ. Grenoble Alpes, CEA, CNRS, 71 Avenue des Martyrs, 38000 Grenoble, France; 2 Physiology Research Center, Iran University of Medical Sciences, Tehran, Iran; 3 Department of Pharmacology, School of Medicine, Iran University of Medical Sciences, Tehran, Iran; 4 Department of Physiology, School of Medicine, Iran University of Medical Sciences, Tehran, Iran

**Keywords:** H9C2 cells, Inflammation, Ischemia/Reperfusion injury, JAK2/STAT3, Lavender oil, p-ERK/ERK

## Abstract

**Objective(s):**

This study was conducted to examine the therapeutic effects of lavender oil (LO) against oxygen-glucose deprivation (OGD)-induced injury* in vitro* model.

**Materials and Methods:**

In this study, the OGD model was induced in the H9C2 cell line, and then the cells were treated with LO (10, 100, 1000, and 10000 μg/ml). The anti-inflammatory activity of LO (JAK2/STAT3) was evaluated by immunocytochemical assay. Likewise, the p-ERK/ERK level was measured by western blotting.

**Results:**

Compared with only the OGD-induced injury model, cell survival increased after treatment with LO. Our results showed that 100 μg/ml of LO significantly decreased the expression of Jak2/Stat3 and the apoptotic activity 72 hr after reperfusion compared with the control group. Likewise, significant increases were observed in p-ERK/ERK in LO-treated groups.

**Conclusion:**

Collectively, these findings confirm that LO can be a good candidate to reduce OGD-induced injury in the H9C2 cell line through targeting Jak2/Stat3 and ERK pathways.

## Introduction

Myocardial ischemia is one of the main reasons for mortality and disability worldwide (1). Even though reperfusion is one of the treatments for ischemia, it might aggravate injury (2). Since reperfusion injury is in the early minutes of reflow, post-conditioning should be at the initial time of reperfusion (3). Despite extensive research about the reduction of MI/R injury, no effective treatment has yet been discovered . 

Extracellular signal-regulated kinase (ERK) is a member of the mitogen-activated protein kinases (MAPKs) family. They are the most ideal candidates among the protein kinases that regulate the cellular transcriptional activities, cell proliferation, differentiation, cell survival, and apoptosis inhibition. ERK1/2 cascade has been revealed to have a vital role in myocardial protection against ischemia-reperfusion injury (4). Many studies have shown that normal myocardial cells possess a low expression of ERK protein expression whereas its expression and activation (phospho-ERK) is markedly increased in myocardial ischemia-reperfusion injury (5). 

Along with ERK1/2 cascade, the Janus kinase/signal transducer and activator of transcription (JAK/STAT) pathway is responsive to cellular signals such as cytokines, growth factors, and hormones and also plays an implicated role in mediating of cardiac pathophysiological processes including hypertrophy, apoptosis, oxidative stress, myocardial interstitial fibrosis, and myocardial infarction signaling (6-9). Current studies have revealed that JAK/STAT, especially JAK2/STAT3 signaling plays an important role in I/R injury (10).

Lavender, which is a member of the Labiatae family, is traditionally used as a plant extract for therapeutic objectives in herbal medicine (11). Lavender essential oil (LEO), is derived from the flowers of Lavandula angustifolia. It consists of many active elements such as linalyl acetate, linalool, 1,8-cineol, lavandulol, lavandulyl acetate, camphor, cis-β-ocimene, trans-β-ocimene, 1-terpinen-4-ol, α-terpineol, limonene, tannins, coumarins, flavonoids, phytosterols, and triterpenes (12). Previous studies have shown that its main ingredients like linalool and linalyl acetate displayed potent anti-oxidant, anti-inflammatory, and antibacterial properties (13). 

In this study, we investigated the post-treatment protective effects of LO against OGD-induced injury *in vitro* model. In addition, we examined the effect of LO on the activities of ERK and JAK2 / STAT3 signaling in the OGD-induced injury model in H9c2 cell lines.

## Materials and Methods

***Materials and chemicals***

IHCplus™ Polyclonal Rabbit antiHuman STAT3 Antibody (aa683732, IHC, WB) LSB4693 was purchased from LifeSpan BioSciences. JAK2 Polyclonal Antibody (bs-23004R) was supplied by Bioss Inc. Immobilon®-FL PVDF membrane pore size 0.45 μm was purchased from Sigma (St. Louis, MO, USA). Dulbecco’s modified Eagle’s medium (DMEM) supplemented with 10% (v/v) inactivated fetal bovine serum (FBS) was obtained from Gibico Company (Carlsbad, CA, USA). ERK1/2 antibody (MBS820689) was obtained from MyBioSource Company. 

***Cell culture***

Embryonic rat myocardium-derived cells (H9c2 cardiomyocytes) were purchased from the Cell Bank of Pasteur Institute (Tehran, Iran) and were used to create the OGD model *in vitro* (14)*. * H9c2 cells were grown in DMEM supplemented with 10% (v/v) inactivated FBS (both from GIBCO), 100 U/ml of penicillin/streptomycin (Aria cell). They were incubated at 37 °C in a humidified atmosphere with 5% CO_2_. Cells were passaged every 3 to 4 days.

***I/R injury model and drug treatment***

Cells were seeded in 6 groups: control group, OGD group (ISC), and four OGD groups treated with different doses (10, 100, 1000, and 10000 μg/ml) of LO. To establish an OGD-induced injury model in H9c2 cell lines, we simultaneously removed nutrients and oxygen from the cells. So, serum-free and glucose-free DMEM were added instead of the culture medium. Cells were incubated in hypoxic conditions (5% CO_2_, 94% N_2_, and 0.5% O_2_) at 37 °C for 4 hr (15). Then, for reperfusion purposes, cells were exposed to DMEM with 10% FBS in an appropriate environment (5% CO_2_ and 37 °C). During re-oxygenation, different concentrations of LO (10, 100, 1000, and 10000 µg/ml) were added to cells in order to investigate the effects of LO on OGD-induced injury.

***MTT assay for cell viability***

MTT assay was done based on previous studies (16, 17). H9C2 cells were plated in 96-well plates at 5×10^3^ cells/well. After 24 hr, for treatment groups, cells were exposed to OGD treatment. In the case of control groups, cells were maintained in the incubator. 24, 48, and 72 hr after treatment, cell viability was evaluated with MTT (3-(4, 5-dimethylthiazol-2-yl)-2, 5-diphenyltetrazolium bromide) assay kit. Briefly, MTT reagent (Sigma, 5 mg/ml) was administered into each well and incubated for 4 hr at 37 °C. Then, dimethyl sulfoxide (DMSO, 100 μl/well) was used to dissolve the formazan crystals . Finally, the absorbance was measured with a microplate reader at 570 nm. The viability of cells was expressed as a percentage of the control and the test was repeated eight times.

***Apoptosis assay***

Cells were cultured at a density of 10^5^ cells/well in 6 well cell culture plates. The experimental groups were exposed to OGD and then treated with LO (10 and 100 μg/ml) after 48 hr, harvested, and washed twice with cold PBS. The cell pellets were resuspended in 1X binding buffer and 5 µl of FITC-labeled annexin V solution was add for 10 min at room temperature (18). Then, 5 µl of PI was added and analyzed by flow cytometry (eBioscience: Thermo Fisher Scientific, USA) and Cell Quest software (Becton-Dickinson) within 4 hr.

***Western blot analyses***

Western blot was done based on a previous study (18). H9C2 cells were collected and washed with cold PBS twice. Cells were homogenized in lysis buffer (100 mM Tris-HCl [pH 8.0], 150 mM NaCl, 0.1% SDS and 1%TritonX-100) containing protease inhibitors on ice. The cell lysate was centrifuged at 1200 RPM for 20 min at 4 °C and the supernatant was gathered. From each group, equal amounts of protein were prepared and loaded on SDS-PAGE gel and separated with electrophorese. The separated bands were transferred onto PVDF membranes, following sealing with 5% skim milk at room temperature for 2 hr. Then, the membranes were exposed to the indicated primary antibodies ERK (1:1000 in TBST), p-ERK (1:1000 in TBST), and β-actin (1:1000 in TBST) at 4 °C overnight. After fully washing with PBS three times, the membrane was probed with secondary antibodies (1:3000 in TBST) at 37 °C for 1 hr. As described above, the samples were washed three times again. Finally, protein bands were visualized by chemiluminescence (ECL, Thermo Scientific, Shanghai, China). And the grayscale was analyzed with the Image Lab software system (Bio-Rad, Hercules, CA, USA). The results were normalized compared with β-actin as an internal reference.

***Immunocytochemical staining***

Immunocytochemical staining was done based on a previous study (19). Cells were seeded in 24-well plates with coverslips at 5 × 10^4 ^per well. On the following day, cells were exposed to OGD, as described above, and then treated with 10 and 100 μg/ml of LO. After 48 hr the cells in each well were gently washed with PBS at room temperature. Then, the wells were incubated in freshly prepared 4% paraformaldehyde-PBS at room temperature for 10 min. Next, they were permeabilized for 5 min with 0.5% Triton X-100 in PBS at RT. Following blocking wells in 1–5% normal serum for 1 hr, cells were incubated with the primary antibody dilution at 4 °C overnight. Afterward, appropriate dilution of fluorochrome-conjugated secondary antibody in 1% normal serum was added and incubated for 1 hr. Afterward, the washing process was completed; the cells were counterstained with DAPI to stain the nuclei. Then, mounting media containing a fluorescence antifade agent was added into the wells. Lastly, the cells were observed under a fluorescence microscope.

***Statistical analysis***

Results were expressed as mean ± SEM. Comparisons between groups were performed using analysis of variance (ANOVA) using GraphPad Prism software. *P*<0.05 was considered to indicate a statistically significant difference.

## Results

***Viability assay of different doses of LO following OGD-induced injury***

**Figure 1:**
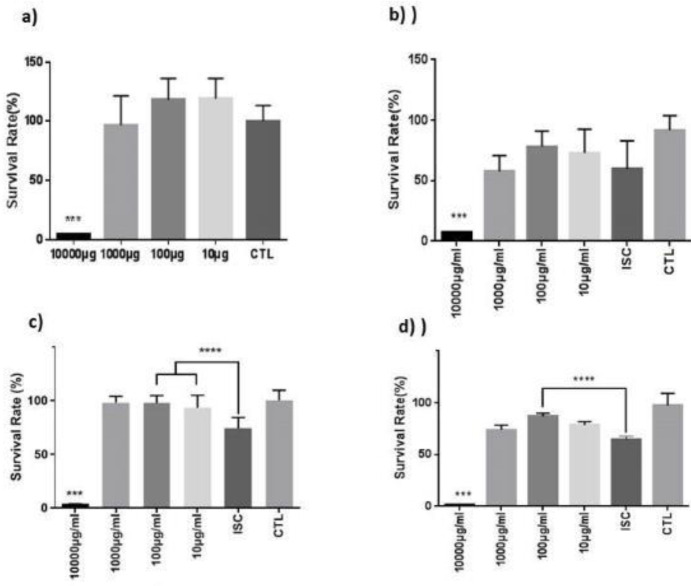
Cell survival rate. a) Effect of LO on normal cells. b) Effects of different dosages of LO on reducing OGD-induced injury after 24 hr. c) Effects of different dosages of LO on reducing OGD-induced injury after 48 hr. d) Effects of different dosages of LO on reducing OGD-induced injury after 72 hr (***P<0.001 vs ISC)

LO: Lavender Oil; OGD: Oxygen-glucose deprivation

As shown in Figure 1a, LO had a cytotoxic effect on normal cells in 10000 μg/ml, and other dosages of LO did not have a significant effect on OGD-induced injury after 24 hr (Figure 1b). Using 100 μg/ml of LO resulted in a %97.5 and %87 survival rate after 48 hr and 72 hr, respectively (Figures 1c and 1d). The viability was significantly enhanced at 100 μg/ml and 48 hr after OGD compared with the untreated groups.

***ICC evaluation of JAK2/STAT3 pathway following OGD-induced injury in H9c2 cardiomyocytes***

**Figure 2:**
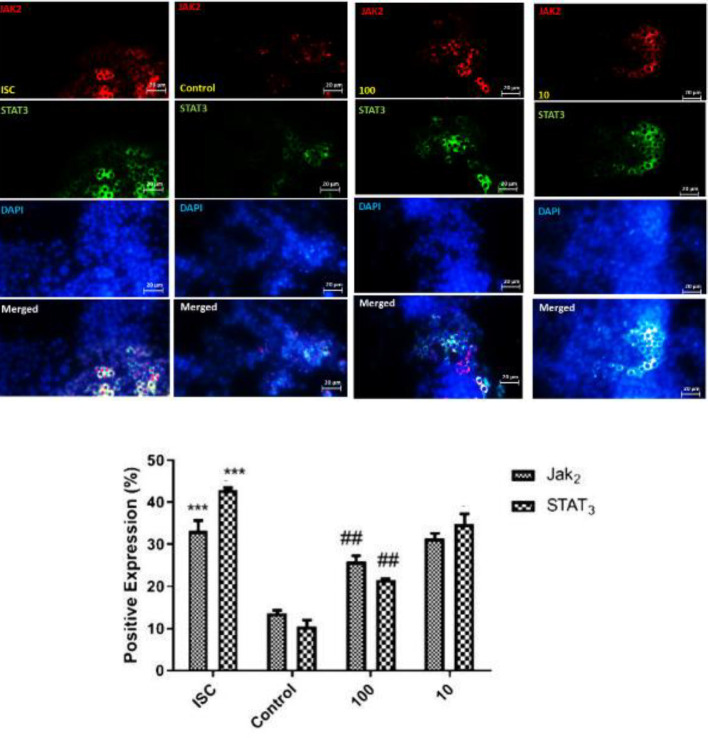
Expression of Jak2/Stat3 in immunocytochemical staining was significantly decreased after treatment compared with the OGD-induced injury (ISC) group (***P<0.001 vs control; ##*P*<0.01 vs ISC)

Expression levels of JAK2/STAT3 were investigated by immunocytochemical analysis in order to assess the effects of LO on this pathway. Compared with the ISC group, post-treatment with LO at the concentrations of 10 and 100 μg/ml significantly decreased the expression levels of STAT3 and JAK2 (Figure 2). The expression rate of JAK2 after 48 hr in OGD cells compared with control cells fell gradually to 33.23% and 13.48%, respectively, and STAT3 expression rate also fell to 42.86% and 10.33% , respectively. In addition to the control groups, in 100 and 10 μg/ml LO post-treatment, the expression of JAK2/STAT3 was significantly less than in the SCI group. 

***LO prevented apoptosis following OGD-induced injury in H9c2 cardiomyocytes***

**Figure 3:**
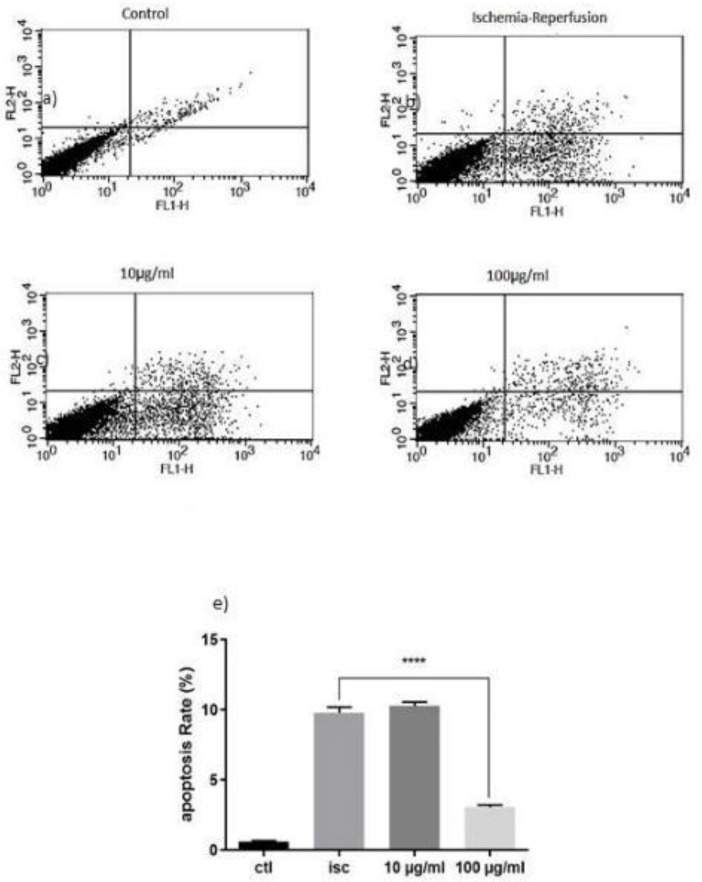
Apoptosis rate 48 hr after treatment in a) Control group, b) OGD (ISC) group, c) LO 10 µg/ml, d) LO 100 µg/ml, and e) Percentage of apoptotic cells (***P<0.001 vs ISC)

OGD: Oxygen-glucose deprivation; LO: Lavender Oil;

To determine whether inhibition of apoptosis in various concentrations of LO changes or not, the percentage of apoptotic cells was measured in the presence of LO using flow cytometric assay, annexin V-FITC, and PI double staining. When cells were exposed to OGD-induced injury, the number of surviving cells was decreased after 48 hr compared with the control group (Figures 3a, 3b). The percentage of apoptotic cells was 9.39% in OGD (ISC) group. While the 100 μg/ml of LO decreased the apoptotic cells to 3.23% (Figures 3d, 3e). These results revealed that LO post-treatment alleviated cardiomyocyte apoptosis.

***LO Treatment Triggers Phosphorylation of ERK***

**Figure 4.**
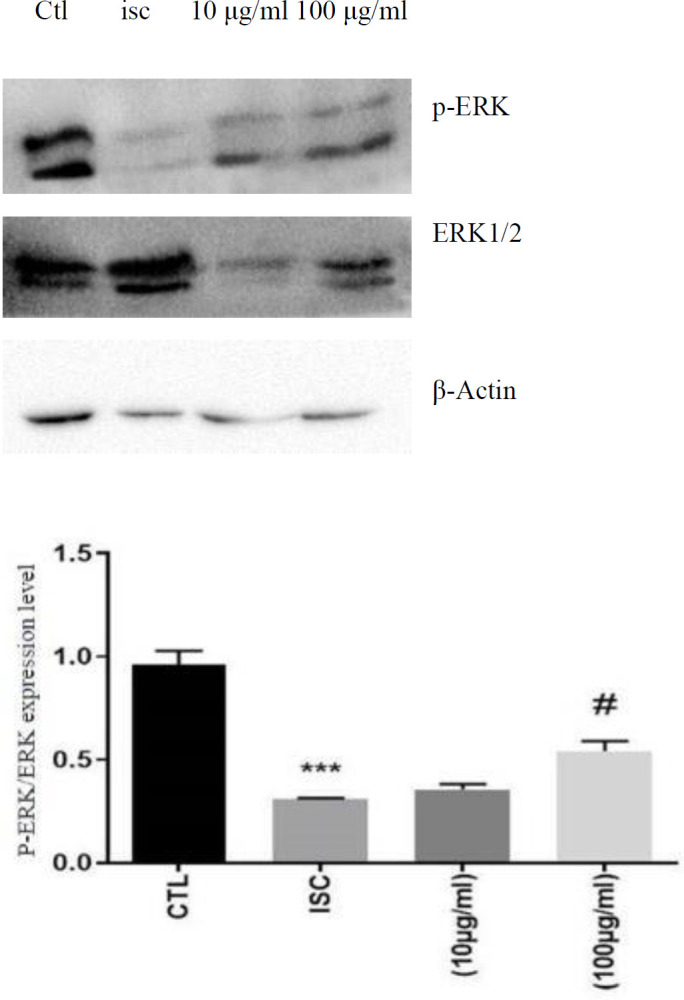
Expression of p-ERK/ERK1.2. Representative western blots showing the expression of p-ERK, ERK1.2, and actin in H9C2 after 48 hr of treatment with LO. Quantification of P-ERK and ERK1/2 expression in different control and treatment groups (***P<0.001 vs control; (#*P*<0.05 vs ISC)

 As shown in Figure 4, the p-ERK protein expression significantly reduced in OGD (ISC) compared with the control group (*P*<0.001). The densitometry scanning confirmed that LO induced phosphorylation of ERK to ERK1/2 in the cardiomyocyte ischemia after 48 hr of treatment compared with untreated groups (Figure 4). Results revealed that LO can considerably stimulate the expression of p-ERK and hinder cardiomyocyte apoptosis.

Jak2/ Stat3: Janus kinases 2/ Signal transducer and activator of transcription proteins 3; OGD: Oxygen-glucose deprivation

ERK: Extracellular Signal-regulated Kinases

## Discussion

In the current study, we used the OGD-induced injury *in vitro* model to explore the protective effect of LO in H9c2 cells and further demonstrate the underlying protective mechanisms. We found that LO leads to cardioprotection against OGD-induced injury in H9c2 cardiomyocytes through inhibition of apoptosis. Outstandingly, it has confirmed the critical role of JAK2/STAT3 signaling in this protective process. This study gives better consideration to the pharmacology of LO in the treatment of OGD-induced injury. Janus kinase (JAK)-signal transducers and activators of transcription (STAT) pathway as a complex signaling network consist of multiple kinases and transcription factors modulating gene expression, thereby might contribute to aggravation of cardio injury in acute myocardial infarction through activation of inflammation (20). Inflammation can play an important role in aggravation of cardiovascular and other diseases (21). 

Previous studies have also shown that JAK2/STAT3 signaling is involved in aggravation of apoptosis in cardiac cells via activation of inflammation (22). Similarly, other researchers demonstrated that JAK2/STAT3 signaling was effectively up-regulated during myocardial ischemia and aggravated injury (23). Here, we showed that JAK2/STAT3 signaling is involved in the protective effects of LO against OGD-induced injury in H9c2 cells. OGD-induced injury significantly increased the phosphorylation of JAK2 and STAT3, whereas this effect was reversed by treatment with LO, indicating the targeting of JAK2/STAT3 signaling by LO in I/R injury. 

ERK1/2 is one of the members of the MAPK signaling pathway, which has an important role in gene transcription and cell cycle processes (24). It promotes both cell survival by inhibition of apoptotic signaling pathways and apoptosis by activation of pro-apoptotic signaling pathways depending on the activated signaling molecules downstream (25, 26). According to the traditional view, phosphorylated ERK enters into the nucleus and regulates the activity of Bcl-2 family members by promoting phosphorylation of the pro-apoptotic protein Bax, inhibiting the activation of pro-apoptotic factors, such as caspase-8 and caspase-3 and release of cytochrome C, eventually keeping cell homeostasis, activating cell survival and proliferation, and inhibiting apoptosis (27, 28). Similarly, other researchers demonstrated that ERK protects against ischemia-reperfusion injury by reducing myocyte apoptosis and infarct size (29, 30). In conclusion, the present study demonstrated the new role of JAK2/STAT3 and ERK pathways in the protective effects of LO against OGD-induced injury in H9c2 cells. 

## Conclusion

This study confirmed that LO by targeting JAK2/STAT3 and ERK-P signaling pathways reduces myocardial apoptosis. These findings may provide new mechanistic insights into the protective roles of LO against OGD-induced injury in H9c2 cells. 

## Funding

This project was supported by Iran University of Medical Sciences. [no. 97-4-32-13301].

## Authors’ Contributions

NA Supervised and conceived the original idea, verified the analytical methods, and checked the whole procedure and paper. SHA, HF, and GV Did the research, analyzed the data, and wrote the paper. FR Performed the western blot experiment. All authors have read and approved the paper.

## Data Availability

The data that support the findings of this study are available from the first author, Shaghayegh Askarian-Amiri, upon reasonable request.

## Conflicts of Interest

All authors declare that there are no conflicts of interest.
